# Ethyl 2-amino-4,5-dimethyl­thio­phene-3-carboxyl­ate

**DOI:** 10.1107/S1600536812026268

**Published:** 2012-06-16

**Authors:** Mostafa M. Ghorab, Mansour S. Al-Said, Hazem A. Ghabbour, Tze Shyang Chia, Hoong-Kun Fun

**Affiliations:** a Medicinal, Aromatic and Poisonous Plants Research Center (MAPPRC), College of Pharmacy, King Saud University, PO Box 2457, Riyadh 11451, Saudi Arabia; b Department of Pharmaceutical Chemistry, College of Pharmacy, King Saud University, PO Box 2457, Riyadh 11451, Saudi Arabia; c X-ray Crystallography Unit, School of Physics, Universiti Sains Malaysia, 11800 USM, Penang, Malaysia

## Abstract

In the title compound, C_9_H_13_NO_2_S, the mean planes of thio­phene ring [maximum deviation = 0.0042 (10) Å] and eth­oxy­carbonyl group [0.0242 (15) Å] are almost coplanar [dihedral angle between them = 0.68 (11)°]. The H atoms of the two methyl groups attached to the thio­phene ring are each disordered over two orientations with site-occupancy ratios of 0.77 (4):0.23 (4) and 0.84 (4):0.16 (4). An intra­molecular N—H⋯O hydrogen bond generates an *S*(6) ring motif. In the crystal, mol­ecules are linked by N—H⋯O hydrogen bonds into an infinite wave-like chain running parallel to the *b*-axis direction. The crystal structure also features C—H⋯π inter­actions.

## Related literature

For the synthesis, see: Gewald (1965) ▶. For background to biologically active compounds prepared from the title compound, see: Alqasoumi *et al.* (2009 ▶); Ghorab *et al.* (2006, ▶ 2012 ▶). For hydrogen-bond motifs, see: Bernstein *et al.* (1995 ▶).
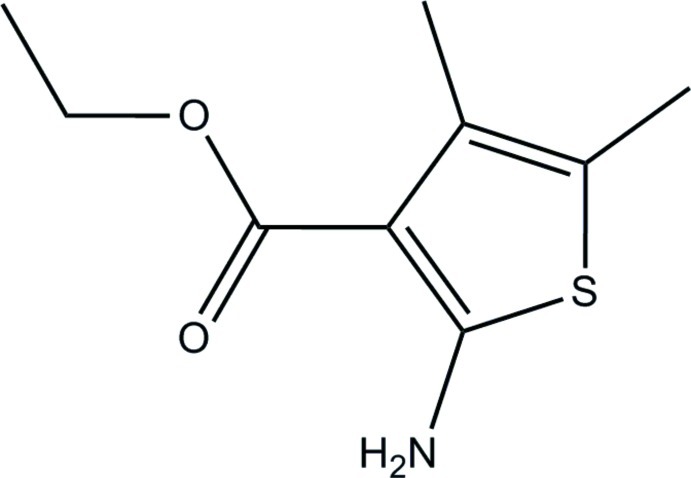


## Experimental

### 

#### Crystal data

C_9_H_13_NO_2_S
*M* *_r_* = 199.26
Monoclinic, 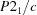

*a* = 7.9487 (2) Å
*b* = 9.8939 (3) Å
*c* = 13.4348 (4) Å
β = 106.143 (2)°
*V* = 1014.90 (5) Å^3^
*Z* = 4
Cu *K*α radiation
μ = 2.59 mm^−1^
*T* = 296 K
0.92 × 0.26 × 0.08 mm

#### Data collection

Bruker SMART APEXII CCD diffractometer
Absorption correction: multi-scan (*SADABS*; Bruker, 2009 ▶) *T*_min_ = 0.199, *T*_max_ = 0.820
6429 measured reflections
1671 independent reflections
1504 reflections with *I* > 2σ(*I*)
*R*_int_ = 0.029

#### Refinement

*R*[*F* ^2^ > 2σ(*F* ^2^)] = 0.037
*wR*(*F* ^2^) = 0.104
*S* = 1.07
1671 reflections
132 parameters
H atoms treated by a mixture of independent and constrained refinement
Δρ_max_ = 0.19 e Å^−3^
Δρ_min_ = −0.17 e Å^−3^

### 

Data collection: *APEX2* (Bruker, 2009 ▶); cell refinement: *SAINT* (Bruker, 2009 ▶); data reduction: *SAINT*; program(s) used to solve structure: *SHELXTL* (Sheldrick, 2008 ▶); program(s) used to refine structure: *SHELXTL*; molecular graphics: *SHELXTL*; software used to prepare material for publication: *SHELXTL* and *PLATON* (Spek, 2009 ▶).

## Supplementary Material

Crystal structure: contains datablock(s) global, I. DOI: 10.1107/S1600536812026268/hb6845sup1.cif

Structure factors: contains datablock(s) I. DOI: 10.1107/S1600536812026268/hb6845Isup2.hkl

Supplementary material file. DOI: 10.1107/S1600536812026268/hb6845Isup3.cml

Additional supplementary materials:  crystallographic information; 3D view; checkCIF report

## Figures and Tables

**Table 1:** Hydrogen-bond geometry (Å, °) *Cg*1 is the centroid of S1/C1–C4 ring.

*D*—H⋯*A*	*D*—H	H⋯*A*	*D*⋯*A*	*D*—H⋯*A*
N1—H1*N*1⋯O2	0.89 (3)	2.06 (3)	2.744 (2)	133 (2)
N1—H2*N*1⋯O2^i^	0.87 (2)	2.12 (2)	2.972 (2)	167 (2)
C8—H8*A*⋯*Cg*1^ii^	0.97	2.78	3.600 (2)	142

Symmetry codes: (i) 
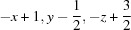
; (ii) 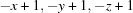.

**Fig. 1.**
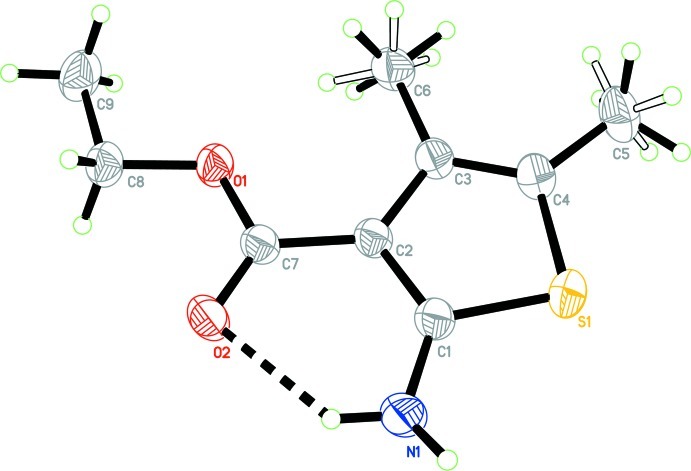
The molecular structure of the title compound with 30% probability displacement ellipsoids. The dashed line represents the intramolecular N—H···O hydrogen bond.

**Fig. 2.**
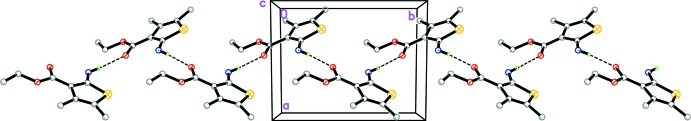
The crystal packing of the title compound. The dashed lines represent the hydrogen bonds. For clarity sake, hydrogen atoms not involved in hydrogen bonding have been omitted.

## supplementary crystallographic information

### Comment

Ethyl 2-amino-4,5-dimethylthiophene-3-carboxylate (Gewald, 1965) is useful in the synthesis of heterocyclic compounds, especially thienopyrimidine derivatives (Alqasoumi *et al.*, 2009), some of which possess biological activities (Ghorab *et al.*, 2006). In the light of this, and as a continuation of our efforts towards synthesizing biologically active heterocyclic compounds (Ghorab *et al.*, 2012), the title compound was prepared and its crystal structure is now reported.

The molecular structure of the title compound is shown in Fig. 1. The mean plane of thiophene ring [S1/C1–C4; maximum deviation = 0.0042 (10) Å at atom C4] is almost coplanar with the mean plane of ethoxycarbonyl group [O1/O2/C7–C9; maximum deviation = 0.0242 (15) Å at atom C8] as indicated by the dihedral angle of 0.68 (11)°. The H atoms attached to atoms C5 and C6 are each disordered over two orientations with site-occupancy ratios of 0.77 (4):0.23 (4) and 0.84 (4):0.16 (4), respectively. An intramolecular N1—H1N1···O2 hydrogen bond generates an S(6) ring motif (Bernstein *et al.*, 1995) in the molecule.

In the crystal (Fig. 2), molecules are linked by N1—H2N1···O2 hydrogen bond into an infinite wave-like chain, propagating along the *b* axis. The crystal packing also features C—H···π interactions (Table 1), involving *Cg*1 which is the centroid of S1/C1–C4 ring.

### Experimental

Ethyl 2-amino-4,5-dimethylthiophene-3-carboxylate was prepared according to the reported method (Gewald, 1965). The obtained solid was recrystallized from ethanol to give the title compound. Brown plates were obtained by slow evaporation from ethanol solution at room temperature.

### Refinement

The atoms H1N1 and H2N1 were located in a difference fourier map and refined freely [N—H = 0.88 (3) and 0.87 (2) Å]. The major parts of disordered H atoms attached to atoms C5 and C6 [(H5A, H5B, H5C) and (H6A, H6B, H6C)] were positioned geometrically, whereas the corresponding minor parts, (H5D, H5E, H5F) and (H6D, H6E, H6F) were located in a difference fourier map. A rotating group model (AFIX 137) was used for both major and minor parts of disordered methyl groups and refined using a riding model with *U*_iso_(H) = 1.5*U*_eq_(C) [C—H distance = 0.96 Å]. The refined site-occupancy ratios are (H5A, H5B, H5C):(H5D, H5E, H5F) = 0.77 (4):0.23 (4) and (H6A, H6B, H6C):(H6D, H6E, H6F) = 0.84 (4):0.16 (4). The remaining H atoms were positioned geometrically [C—H = 0.96 and 0.97 Å] and refined with *U*_iso_(H) = 1.2 or 1.5*U*_eq_(C). A rotating group model was also applied to the other methyl group in the final refinement.

### Crystal data

**Table:**

C_9_H_13_NO_2_S	*F*(000) = 424
*M**_r_* = 199.26	*D*_x_ = 1.304 Mg m^−^^3^
Monoclinic, *P*2_1_/*c*	Cu *K*α radiation, λ = 1.54178 Å
Hall symbol: -P 2ybc	Cell parameters from 1386 reflections
*a* = 7.9487 (2) Å	θ = 3.4–70.2°
*b* = 9.8939 (3) Å	µ = 2.59 mm^−^^1^
*c* = 13.4348 (4) Å	*T* = 296 K
β = 106.143 (2)°	Plate, brown
*V* = 1014.90 (5) Å^3^	0.92 × 0.26 × 0.08 mm
*Z* = 4	

### Data collection

**Table:**

Bruker SMART APEXII CCD diffractometer	1671 independent reflections
Radiation source: fine-focus sealed tube	1504 reflections with *I* > 2σ(*I*)
Graphite monochromator	*R*_int_ = 0.029
φ and ω scans	θ_max_ = 65.0°, θ_min_ = 5.6°
Absorption correction: multi-scan (*SADABS*; Bruker, 2009)	*h* = −6→8
*T*_min_ = 0.199, *T*_max_ = 0.820	*k* = −11→11
6429 measured reflections	*l* = −15→15

### Refinement

**Table:**

Refinement on *F*^2^	Secondary atom site location: difference Fourier map
Least-squares matrix: full	Hydrogen site location: inferred from neighbouring sites
*R*[*F*^2^ > 2σ(*F*^2^)] = 0.037	H atoms treated by a mixture of independent and constrained refinement
*wR*(*F*^2^) = 0.104	*w* = 1/[σ^2^(*F*_o_^2^) + (0.0573*P*)^2^ + 0.1221*P*] where *P* = (*F*_o_^2^ + 2*F*_c_^2^)/3
*S* = 1.07	(Δ/σ)_max_ = 0.001
1671 reflections	Δρ_max_ = 0.19 e Å^−^^3^
132 parameters	Δρ_min_ = −0.17 e Å^−^^3^
0 restraints	Extinction correction: *SHELXTL* (Sheldrick, 2008), Fc^*^=kFc[1+0.001xFc^2^λ^3^/sin(2θ)]^-1/4^
Primary atom site location: structure-invariant direct methods	Extinction coefficient: 0.0041 (9)

### Special details

**Table:**

Geometry. All e.s.d.'s (except the e.s.d. in the dihedral angle between two l.s. planes) are estimated using the full covariance matrix. The cell e.s.d.'s are taken into account individually in the estimation of e.s.d.'s in distances, angles and torsion angles; correlations between e.s.d.'s in cell parameters are only used when they are defined by crystal symmetry. An approximate (isotropic) treatment of cell e.s.d.'s is used for estimating e.s.d.'s involving l.s. planes.
Refinement. Refinement of *F*^2^ against ALL reflections. The weighted *R*-factor *wR* and goodness of fit *S* are based on *F*^2^, conventional *R*-factors *R* are based on *F*, with *F* set to zero for negative *F*^2^. The threshold expression of *F*^2^ > σ(*F*^2^) is used only for calculating *R*-factors(gt) *etc*. and is not relevant to the choice of reflections for refinement. *R*-factors based on *F*^2^ are statistically about twice as large as those based on *F*, and *R*- factors based on ALL data will be even larger.

### Fractional atomic coordinates and isotropic or equivalent isotropic displacement parameters (Å^2^)

**Table:**

	*x*	*y*	*z*	*U*_iso_*/*U*_eq_	Occ. (<1)
S1	0.19746 (6)	0.19752 (4)	0.58473 (4)	0.0611 (2)	
O1	0.31318 (16)	0.65860 (11)	0.49116 (9)	0.0545 (3)	
O2	0.44541 (19)	0.61171 (12)	0.65701 (9)	0.0667 (4)	
N1	0.3976 (3)	0.35938 (18)	0.72936 (12)	0.0726 (5)	
C1	0.3045 (2)	0.34698 (16)	0.62875 (12)	0.0520 (4)	
C2	0.2743 (2)	0.44243 (15)	0.55082 (11)	0.0458 (4)	
C3	0.1625 (2)	0.39154 (16)	0.45331 (12)	0.0481 (4)	
C4	0.1130 (2)	0.26238 (18)	0.46019 (14)	0.0554 (4)	
C5	0.0030 (3)	0.1727 (2)	0.37724 (19)	0.0763 (6)	
H5A	0.0682	0.1482	0.3296	0.114*	0.77 (4)
H5B	−0.1014	0.2200	0.3408	0.114*	0.77 (4)
H5C	−0.0282	0.0926	0.4083	0.114*	0.77 (4)
H5D	−0.0262	0.2197	0.3122	0.114*	0.23 (4)
H5E	−0.1025	0.1489	0.3945	0.114*	0.23 (4)
H5F	0.0673	0.0922	0.3720	0.114*	0.23 (4)
C6	0.1078 (2)	0.46993 (19)	0.35388 (13)	0.0622 (5)	
H6A	0.0388	0.4131	0.2999	0.093*	0.84 (4)
H6B	0.2100	0.5001	0.3355	0.093*	0.84 (4)
H6C	0.0396	0.5468	0.3627	0.093*	0.84 (4)
H6D	−0.0103	0.4465	0.3171	0.093*	0.16 (4)
H6E	0.1843	0.4485	0.3120	0.093*	0.16 (4)
H6F	0.1145	0.5650	0.3689	0.093*	0.16 (4)
C7	0.3521 (2)	0.57551 (15)	0.57265 (11)	0.0469 (4)	
C8	0.3871 (3)	0.79279 (16)	0.50551 (15)	0.0568 (4)	
H8A	0.5139	0.7884	0.5241	0.068*	
H8B	0.3524	0.8395	0.5602	0.068*	
C9	0.3179 (3)	0.8652 (2)	0.40444 (17)	0.0718 (6)	
H9A	0.3617	0.9561	0.4109	0.108*	
H9B	0.1923	0.8668	0.3862	0.108*	
H9C	0.3554	0.8190	0.3515	0.108*	
H1N1	0.453 (3)	0.438 (3)	0.7431 (19)	0.084 (7)*	
H2N1	0.435 (3)	0.291 (2)	0.7699 (19)	0.068 (6)*	

### Atomic displacement parameters (Å^2^)

**Table:**

	*U*^11^	*U*^22^	*U*^33^	*U*^12^	*U*^13^	*U*^23^
S1	0.0783 (4)	0.0381 (3)	0.0662 (3)	−0.00222 (17)	0.0189 (2)	0.00472 (17)
O1	0.0677 (7)	0.0409 (6)	0.0480 (6)	−0.0061 (5)	0.0046 (5)	0.0053 (5)
O2	0.0971 (10)	0.0438 (6)	0.0460 (7)	−0.0060 (6)	−0.0019 (6)	−0.0041 (5)
N1	0.1166 (14)	0.0449 (9)	0.0439 (8)	0.0066 (9)	0.0018 (8)	0.0056 (7)
C1	0.0693 (10)	0.0379 (8)	0.0470 (9)	0.0063 (7)	0.0132 (7)	0.0005 (6)
C2	0.0556 (9)	0.0371 (8)	0.0422 (8)	0.0051 (6)	0.0094 (6)	0.0001 (6)
C3	0.0503 (9)	0.0433 (8)	0.0470 (8)	0.0018 (6)	0.0075 (6)	−0.0021 (6)
C4	0.0558 (10)	0.0467 (9)	0.0603 (10)	−0.0014 (7)	0.0106 (7)	−0.0046 (7)
C5	0.0745 (13)	0.0581 (11)	0.0847 (15)	−0.0130 (9)	0.0031 (10)	−0.0155 (10)
C6	0.0696 (11)	0.0604 (11)	0.0455 (9)	−0.0006 (8)	−0.0024 (7)	0.0013 (7)
C7	0.0578 (9)	0.0382 (8)	0.0414 (8)	0.0048 (6)	0.0081 (6)	0.0001 (6)
C8	0.0671 (11)	0.0406 (9)	0.0592 (10)	−0.0047 (7)	0.0121 (8)	0.0038 (7)
C9	0.0820 (14)	0.0516 (11)	0.0755 (13)	−0.0027 (9)	0.0114 (10)	0.0196 (9)

### Geometric parameters (Å, º)

**Table:**

S1—C1	1.7264 (17)	C5—H5C	0.9600
S1—C4	1.7429 (18)	C5—H5D	0.9600
O1—C7	1.3348 (19)	C5—H5E	0.9600
O1—C8	1.4429 (19)	C5—H5F	0.9600
O2—C7	1.2228 (19)	C6—H6A	0.9600
N1—C1	1.354 (2)	C6—H6B	0.9600
N1—H1N1	0.88 (3)	C6—H6C	0.9600
N1—H2N1	0.87 (2)	C6—H6D	0.9600
C1—C2	1.381 (2)	C6—H6E	0.9600
C2—C7	1.450 (2)	C6—H6F	0.9600
C2—C3	1.453 (2)	C8—C9	1.498 (3)
C3—C4	1.348 (2)	C8—H8A	0.9700
C3—C6	1.501 (2)	C8—H8B	0.9700
C4—C5	1.501 (3)	C9—H9A	0.9600
C5—H5A	0.9600	C9—H9B	0.9600
C5—H5B	0.9600	C9—H9C	0.9600
			
C1—S1—C4	92.01 (8)	H5E—C5—H5F	109.5
C7—O1—C8	117.66 (13)	C3—C6—H6A	109.5
C1—N1—H1N1	112.9 (16)	C3—C6—H6B	109.5
C1—N1—H2N1	123.7 (15)	C3—C6—H6C	109.5
H1N1—N1—H2N1	119 (2)	C3—C6—H6D	109.5
N1—C1—C2	128.80 (17)	C3—C6—H6E	109.5
N1—C1—S1	120.01 (14)	H6D—C6—H6E	109.5
C2—C1—S1	111.16 (12)	C3—C6—H6F	109.5
C1—C2—C7	119.57 (14)	H6D—C6—H6F	109.5
C1—C2—C3	112.36 (14)	H6E—C6—H6F	109.5
C7—C2—C3	128.07 (13)	O2—C7—O1	121.49 (14)
C4—C3—C2	112.56 (14)	O2—C7—C2	124.63 (14)
C4—C3—C6	122.22 (15)	O1—C7—C2	113.88 (13)
C2—C3—C6	125.21 (15)	O1—C8—C9	106.59 (15)
C3—C4—C5	129.10 (18)	O1—C8—H8A	110.4
C3—C4—S1	111.91 (12)	C9—C8—H8A	110.4
C5—C4—S1	118.99 (15)	O1—C8—H8B	110.4
C4—C5—H5A	109.5	C9—C8—H8B	110.4
C4—C5—H5B	109.5	H8A—C8—H8B	108.6
C4—C5—H5C	109.5	C8—C9—H9A	109.5
C4—C5—H5D	109.5	C8—C9—H9B	109.5
C4—C5—H5E	109.5	H9A—C9—H9B	109.5
H5D—C5—H5E	109.5	C8—C9—H9C	109.5
C4—C5—H5F	109.5	H9A—C9—H9C	109.5
H5D—C5—H5F	109.5	H9B—C9—H9C	109.5
			
C4—S1—C1—N1	178.55 (17)	C2—C3—C4—S1	0.56 (19)
C4—S1—C1—C2	0.60 (14)	C6—C3—C4—S1	179.60 (14)
N1—C1—C2—C7	1.8 (3)	C1—S1—C4—C3	−0.67 (15)
S1—C1—C2—C7	179.54 (12)	C1—S1—C4—C5	178.19 (17)
N1—C1—C2—C3	−178.12 (19)	C8—O1—C7—O2	0.8 (2)
S1—C1—C2—C3	−0.40 (18)	C8—O1—C7—C2	−179.18 (15)
C1—C2—C3—C4	−0.1 (2)	C1—C2—C7—O2	0.0 (3)
C7—C2—C3—C4	179.95 (16)	C3—C2—C7—O2	179.97 (16)
C1—C2—C3—C6	−179.12 (16)	C1—C2—C7—O1	−179.94 (14)
C7—C2—C3—C6	0.9 (3)	C3—C2—C7—O1	0.0 (2)
C2—C3—C4—C5	−178.16 (19)	C7—O1—C8—C9	−177.97 (15)
C6—C3—C4—C5	0.9 (3)		

### Hydrogen-bond geometry (Å, º)

*Cg*1 is the centroid of S1/C1–C4 ring.

**Table:**

*D*—H···*A*	*D*—H	H···*A*	*D*···*A*	*D*—H···*A*
N1—H1*N*1···O2	0.89 (3)	2.06 (3)	2.744 (2)	133 (2)
N1—H2*N*1···O2^i^	0.87 (2)	2.12 (2)	2.972 (2)	167 (2)
C8—H8*A*···*Cg*1^ii^	0.97	2.78	3.600 (2)	142

Symmetry codes: (i) −*x*+1, *y*−1/2, −*z*+3/2; (ii) −*x*+1, −*y*+1, −*z*+1.
